# Supplementary material for: Predicting wave overtopping thresholds on coral reef-island shorelines with future sea-level rise
Source: Nat Commun. 2018 Sep 28;9:3997. doi: 10.1038/s41467-018-06550-1 (PMC6162202; doi:10.1038/s41467-018-06550-1)
Supplement: Supplementary file 1 — Supplementary Information [file 41467_2018_6550_MOESM1_ESM.pdf]

Predicting wave overtopping thresholds on coral reef island shorelines with future sea-level rise

Beetham et al.

Supplementary information:

Supplementary Table 1: Page 2

Supplementary Fig. 1: Page 2

Supplementary Fig. 2: Page 3

Supplementary Table 2: Page 4

Supplementary Table 1 | Incident wave and morphology conditions that were iterated to comprise the 60,000 simulations used in this analysis.

Variable	Simulated values									
H_s (m)	0.5	1	1.5	2	2.5	3	3.5	4	4.5	5
T_s (s)	6	8	10	12	14	16				
W_r (m)	50	100	150	200	300	400	500	600	800	1000
h_r (m)	0	0.5	1	1.5	2	2.5	3	3.5	4	4.5
Z_i (m)	0.25	0.5	1	1.5	2	2.5	3	3.5	4	4.5

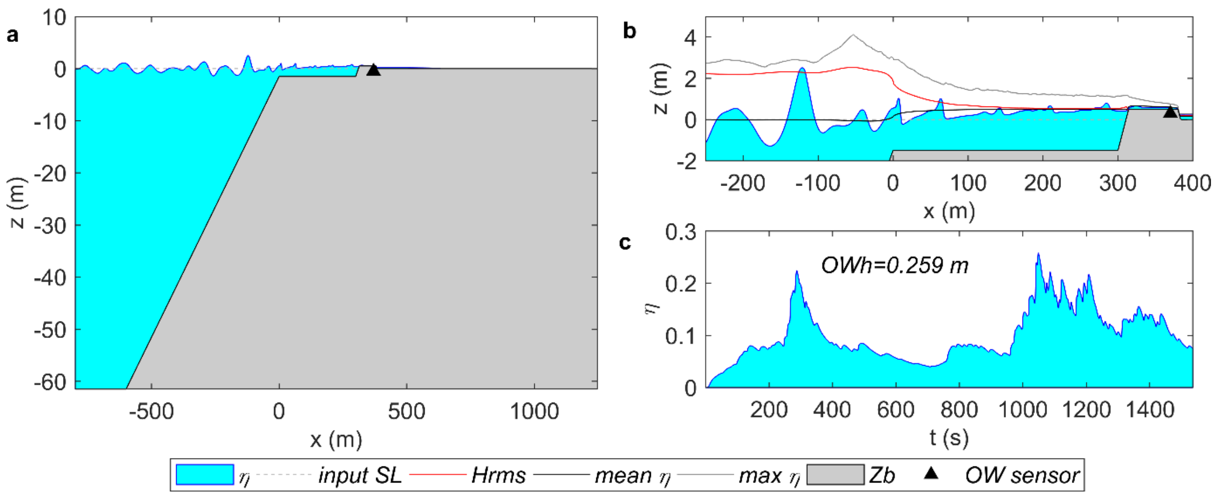

Supplementary Fig. 1 Example model domain and overwash output. Model example with H_s = 3.5 m, T_s = 14 s, h_r = 1.5 m, W_r = 300 m and Z_i = 0.5 m showing **a**, the full model domain and free-surface. **b**, The same output but cropped to highlight wave profiles, wave dissipation (root-mean square wave height, H_{rms}), and water level dynamics on the reef flat and overtopping of the island. **c**, Time-series of overwash flow across the berm.

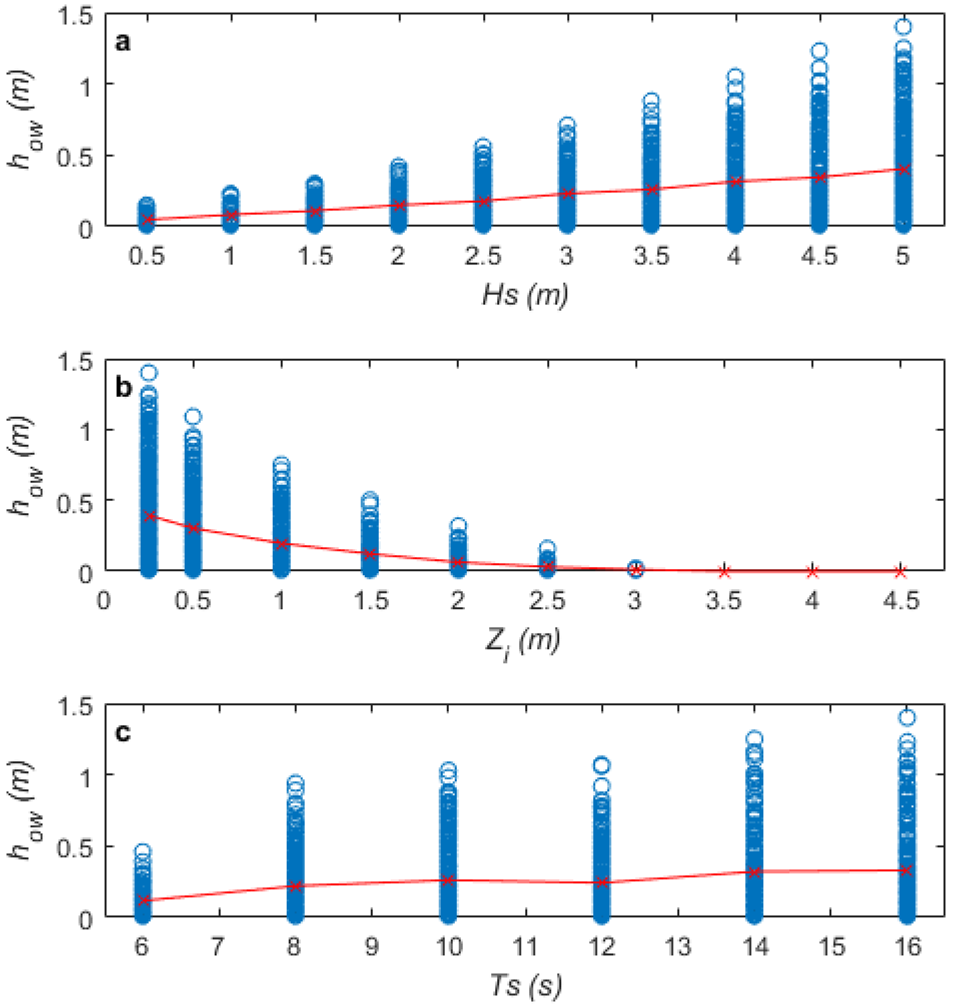

Supplementary Fig. 2 Influence of different variables on overwash depth for a fixed RRW scenario of W_r/h_r = 100. **a**) Incident wave height, **b**) island berm elevation, **c**) incident wave period. Circle markers are shown to represent the maximum overwash depth for each simulation with W_r/h_r = 100 and the red line with cross markers indicates the mean value.

Supplementary Table 2 | Wave climate and morphology characteristics used to assess island inundation vulnerability.

	**Fatato**	**Roi-Namur NW**	**Roi-Namur NE**	**Nukutoa**	**Majuro South**	**Fares Maathoda**
p50 H_s *(m)*	1.27	1.55	1.55	1.18	1.20	1.50
p90 H_s *(m)*	1.72	2.29	2.29	1.59	1.57	2.13
p99 H_s *(m)*	2.19	2.95	2.95	2.02	2.02	2.64
p99.9 H_s *(m)*	2.52	3.71	3.71	2.37	2.62	3.10
Extreme H_s *(m)*	3.11	6.20	6.20	3.31	3.52	3.70
W_r *(m)*	100	270	350	400	90	140
STR	2.00	1.60	1.60	1.75	1.60	0.96
Island characteristics at mean sea level (MSL)						
h_r *(m)*	0.77	0.70	0.70	0.35	0.80	0.30
Z_i *(m)*	3.50	2.80	3.05	1.60	2.30	1.70
RRW	130	386	500	1333	113	467
RIH (*p99*)	1.60	0.95	1.03	0.79	1.14	0.64
Island characteristics at spring high tide (SHT)						
h_r *(m)*	1.77	1.50	1.50	1.23	1.60	0.78
Z_i *(m)*	2.50	2.00	2.25	0.73	1.50	1.22
RRW	56	180	233	340	56	179
RIH (*p99*)	1.14	0.68	0.76	0.36	0.74	0.46
Island characteristics at SHT + 1 m of SLR						
h_r *(m)*	2.77	2.50	2.50	2.18	2.60	1.78
Z_i *(m)*	1.50	1.00	1.25	0.00	0.50	0.22
RRW	36	108	140	184	35	79
RIH (*p99*)	0.68	0.34	0.42	0.00	0.25	0.08
Island characteristics at SHT + 2 m of SLR						
h_r *(m)*	3.77	3.50	3.50	3.18	3.60	2.78
Z_i *(m)*	0.50	0.00	0.25	0.00	0.00	0.00
RRW	27	77	100	126	25	50
RIH (*p99*)	0.23	0.00	0.08	0.00	0.00	0.00
