# Supplementary material for: Predicting wave overtopping thresholds on coral reef-island shorelines with future sea-level rise
Source: Nat Commun. 2018 Sep 28;9:3997. doi: 10.1038/s41467-018-06550-1 (PMC6162202; doi:10.1038/s41467-018-06550-1)
Supplement: Supplementary file 3 — Description of Additional Supplementary Files [file 41467_2018_6550_MOESM3_ESM.docx]

**Description of Additional Supplementary Files**

File Name: Supplementary Data 1

Description: Model outputs for overwash magnitude, with associated input parameters for all 60,000 simulations.

File Name: Supplementary Data 2

Description: Significant overwash magnitude associated with each combination of relative reef width and relative island elevation.
